# Risk Factors Associated with Recurrent Pregnancy Loss and Outcome of Pre-Implantation Genetic Screening of Affected Couples

**DOI:** 10.22074/IJFS.2021.137626.1027

**Published:** 2021-10-16

**Authors:** Nayeralsadat Fatemi, Maryam Varkiani, Fariba Ramezanali, Babak Babaabasi, Azadeh Ghaheri, Alireza Biglari, Mehdi Totonchi

**Affiliations:** 1. Department of Genetics and Molecular Medicine, School of Medicine, Zanjan University of Medical Sciences (ZUMS), Zanjan, Iran; 2. Department of Genetics, Reproductive Biomedicine Research Center, Royan Institute for Reproductive Biomedicine, ACECR, Tehran, Iran; 3. Department of Molecular Genetics, Faculty of Basic Sciences and Advanced Technologies in Biology, University of Science and Culture, Tehran, Iran; 4. Department of Endocrinology and Female Infertility, Reproductive Biomedicine Research Center, Royan Institute for Reproductive Biomedicine, ACECR, Tehran, Iran; 5. Reproductive Epidemiology Research Center, Royan Institute for Reproductive Biomedicine, ACECR, Tehran, Iran; 6. Department of Stem Cells and Developmental Biology, Cell Science Research Center, Royan Institute for Stem Cell Biology and Technology, ACECR, Tehran, Iran

**Keywords:** Array-CGH, Chromosomal Abnormalities, Recurrent Pregnancy Loss

## Abstract

**Background:**

Recurrent pregnancy loss (RPL) is a multifactorial disorder which affects up to 5% of couples around the world. Several factors are considered to be involved in RPL; but, the etiology remains unexplained in 35-60% of cases. The aim of this study was to assess the frequency of risk factors associated with RPL in a group of our clinic clients, and their pre-implantation genetic screening (PGS) outcome.

**Materials and Methods:**

We designed a retrospective descriptive study among, 602 Iranian couples referred to the Royan Reproductive Clinic (Tehran-Iran) from 2006 to 2018. Their karyotyping test and PGS outcomes were analyzed. PGS had been applied by array comparative genomic hybridization (array-CGH) on embryos from these patients. Also, karyotyping test had been performed using standard cytogenetic techniques.

**Results:**

G-banding analysis revealed a frequency of 15.61% chromosomal abnormalities in RPL couples. Also, the reciprocal translocations were more frequent (33/1204 cases) compared to the other structural abnormalities. Pregnancy rate per embryo transferred were 50% with array-CGH approach.

**Conclusion:**

Our findings could confirm a positive correlation between chromosomal abnormalities and RPL rate. Applying PGS for the RPL couples, leads to improvement of pregnancy success rate.

## Introduction

Recurrent pregnancy loss (RPL) is an important and common phenomenon in the reproductive system, which affects 2-5% of couples (1). According to the American Society for Reproductive Medicine (ASRM), RPL is defined as two or more consecutive pregnancy losses before 20 weeks while, minimum of three failed pregnancy (<20 weeks gestation) is determined by European Society of Human Reproduction and Embryology (ESHRE) and the Royal College of Obstetricians and Gynecologists (RCOG) (2). RPL occurrence , a highly heterogeneous condition , was attributed to several causes including endocrine dysfunction, auto immune disorders, thrombophilia, genetic abnormalities, infectious diseases, uterine anomalies, sperm DNA fragmentation and epigenetics (3, 4). However, the reason for half of RPL cases is still unclear (1).

Genetic factors such as chromosomal rearrangements and gene mutations are responsible for 2-5% of the defined causes of RPL (4). Chromosomal balanced structural rearrangements, mainly reciprocal and Robertsonian translocations , were identified more common in couples with recurrent spontaneous abortions (5, 6). Also embryo chromosomal abnormalities such as, aneuploidy and polyploidy, were observed in 50-80% of aborted tissues, which are the most important reason for first-trimester spontaneous pregnancy loss (7). Nowadays, pre-implantation genetic screening (PGS) is performed to improve the in vitro fertilization (IVF) success rate (8) by embryo chromosomal abnormalities detection.

Before advent of array comparative genomic hybridization (array-CGH), fluorescence in situ hybridization (FISH) technique was performed as screening approach for over two decades. Recently, due to FISH limitations, it has been recommended that this technique should be replaced by developed screening methods such as next generation sequencing (NGS) and array-CGH (9). Unlike FISH, array-CGH could analyze all 24 chromosomes and shows high accuracy for aneuploidy detection (10). Recent advances in NGS technology, enable to use this technique for chromosomal screening in preimplantation embryos (11). Moreover, using this technique is challenging due to detecting large insertions and deletions (indels) (>1 kb) and complex structural variations (12).

The present study is to find out the relation between chromosomal abnormality and RPL among patients referred to the Royan Reproductive Clinic (Tehran-Iran) from 2006 to 2018.

## Materials and Methods

### Patients

This retrospective descriptive study includes a total of 1204 individuals (602 couples) with RPL history (more than two consecutive pregnancy losses before 20 weeks of gestation) referring to the Royan Reproductive Clinic, Tehran, Iran, during the period of 2006 to 2018. Informed consent was obtained from all patients according to the Royan Institute Ethical Committee Guidelines. The study was performed in accordance with the Declaration of Helsinki and was approved by Institutional Review Board and Ethics Committees (Royan Institute: IR.ACECR. ROYAN.REC.1397.213, Zanjan University of Medical Sciences: ZUMS.REC.1396.182). Also, the most common RPL causes, such as hysterosalpingography, immunological tests, semen analysis, clotting assay, and blood tests for diabetes mellitus, hypothyroidism and infectious cause history were investigated for each couple.

In this study, severe intrauterine adhesions and Mullerian tract anomalies described as anatomical abnormalities in female reproduction system. Also, we categorized diabetes type II, polycystic ovarian syndrome, hypothyroidism, endometriosis and hyperprolactinemia as endocrine problems Thrombophilic genetic factors such as homozygous mutations in each of the *MTHFR* (C677T), Factor V Leiden (1691G > A), *PAI-1* (4G/4G) and prothrombin (G20210A) genes were reported. According to ESHRE guideline (3), thrombophilia-related mutations were evaluated for the patients who had additional risk factors for thrombophilia or had a family history. Also, sperm DNA fragmentation index (DFI) and high DNA stainability (HDS) were assessed by the sperm chromatin structure assay (SCSA). For the determination of sperm DNA damage, we considered DFI >25% or HDS >15%.

### Cytogenetic analysis

Karyotyping from peripheral blood lymphocytes was performed for both male and female partners, according to standard cytogenetic techniques (12). Briefly, at least 25 metaphase cells were analyzed for each patient while every suspected mosaic cases received extensive work-up, additional cells were examined to exclude 10% mosaicism at a 95% confidence level. Polymorphic rearrangements including heterochromatin variants were considered normal karyotypes. Karyotypes were described according to the International System for Human Cytogenetic Nomenclature criteria (ISCN) (13).

### Pre-implantation genetic screening

Using array-CGH, PGS was performed to identify embryos chromosomal aneuploidy during assisted reproductive technology (ART) treatment. Following the long protocol ovarian stimulation (14), the mature oocytes were fertilized by intracytoplasmic sperm injection (ICSI) and cycles testing of blastomeres was performed in 3-day embryos.

Using array-CGH, single-cell numerical chromosomal abnormalities were verified that those cells removed on day 3 to 5 in early embryo stages. In this aim, the 24 sure ® Microarray Pack version 3.0 (Illumina®; cat. #: PR-10- 408702-PK, USA) was applied.

The array slids was scanned in InnoScan 900 microarray scanner (INNOPSYS Inc., Carbonne, France) and, Data were analyzed using the BlueFuse Multi v3.1 software program (Illumina). Depending on the platform used, BlueFuse Multi software (BlueGnome Ltd, now Illumina) calculates median log 2 ratio for all the chromosomes, as the index of aneuploidy.

## Results

Five hundred forty eight couples out of 602, (91.02%) had a first trimester abortion experience (<13 weeks). Also, the percentage of couples with ≥3 abortion was 78.24% (the average abortion was 3.5 ± 1.6). Karyotype analysis showed 8.13% (98/1204, 73 females and 25 males) chromosomal abnormalities in RPL patients. The reciprocal translocations were more frequent structural abnormality (2.74%) in 602 studied couples. The frequency and types of chromosomal abnormalities are shown in Tables 1 and 2.

**Table 1:** The frequency and types of chromosomal abnormality in 602 couples (1204 cases)


Type	Number	Frequency in 1204 cases (%)

Mosaicism	35	2.90
Translocation	40	3.32
Robertsonian	7	0.58
Reciprocal	33	2.74
Inversion	22	1.82
Super male	1	0.08
Total	99	8.22


Gynecologic structural abnormalities were identified in 16.77% (101/602) of the patients. Endocrine disorder and thrombotic complications were observed in 26.07% (157/602) and 4.15% (25/602) of the females, respectively, while, sperm DNA damage were detected in 14.95% of couples subjected to RPL (Table 3).

### Pre-implantation genetic screening analysis

In this study, only 83 couples (83/602) were undertaken PGS with array-CGH platform. Only the last cycle of PGS was considered for each couple.

**Table 2:** Structural chromosome abnormalities of the carrier couples with recurrent pregnancy loss (RPL)


Structural chromosome abnormalities	Female	Male

Reciprocal translocation	46,XX,t(11;22)(q23;q11.2)	46,XY,t(1;2)(p36.2;q37.2)
	46, XX t(10;15)(q21;q21)	46,XY,t(16;6)(p12;q26)
	46,XX,t(16;6)(p12;q26)	46,XY,t(6;12)(q15;q15)
	46,XX,t(1;3)(q32;q13.2)	46,XY,t(1;13)(q43;q14)
	46,XX,t(5;16)(p15.1;q12.1)	46,XY,t(1;14)(q43;q25)
	46XX,t(1;13)(q21;q12.3)	46,XY,t(7;10)(q21.3;q26.2)
	46,XX,t(13;11)	46,XY,t(18;20)(q12.2;q13.1)
	46,XX,t(11;22)(q23;q11.2)	46,XY,t(1;3)(p35.1;p26)
	46,XX,t(4;12)(q35;q22)	46,XY,t(10;19)(q22;q13)
	46,XX,t(4;7)(q35;q31.2)	46,XY,t(1;7)(q21;q36)
	46,XX,t(2;18)(p24:q2.2)	46,XY,t(13q:16q)
	46,XX,t(6;18)(q25.1;q21.1)	46,XY,t(4;10)(q22;q21)
	46,XX,t(4;7)(q27;p14.1)	46,XY,t(4;8)(q33;q23)
	46,XX,t(3;20)(q13.3;p12)	46,XY,t(4;6)(q26;p24)
	46,XX,t(1;11)(p32.9;p14.3)	46,XY,t(1;15)(p36.1;p11.2)
	46,XX,t(2,3)(q12;q27)	46,XY,t(6;11)(q13;q25)
		46,XY,t(4;5)(p14;q15)
Robertsonian translocation	45,XX,t(13;14)(q10;q10)	46,XY,t(13q:16q)
	45,XX,der(14;15)(q10;q10)	45,XY,t(13,14)
	45,XX,der(14;21)(q10;q10)	
Inversion	46, XX, inv (5)(p13q13)	46,XY, inv(9)(p13q21)
	46,XX,inv(9)(p11q12)	46,XY,per inv(9)(p11q12)
	46,XX,inv(4)(q10q12)	46,XY,inv(11)(p15q13)
	46,XX,per inv(8)(p23.1q22.1)	


**Table 3:** Frequency of factors associated with recurrent pregnancy loss (RPL) in 602 couples


Type		Number	Frequency (%)

Couples with chromosomal abnormality		94	15.61
Anatomical abnormalities in female reproduction system		101	16.77
	Uterine adhesions	86	14.28
	Mullerian tract anomalies	15	2.49
Endocrine disorder in female		157	26.07
	Diabetes type II	20	3.32
	Polycystic ovary syndrome	43	7.14
	Hypothyroidism	97	16.11
	Endometriosis	7	1.16
	Hyperprolactinemia	3	0.49
Thrombotic		25	4.15
Males with sperm DNA damages		90	14.95


**Fig 1:**
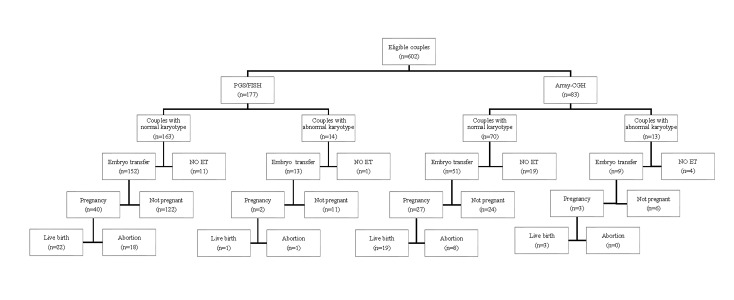
Flowchart of eligible subjects and their outcomes.

**Fig 2:**
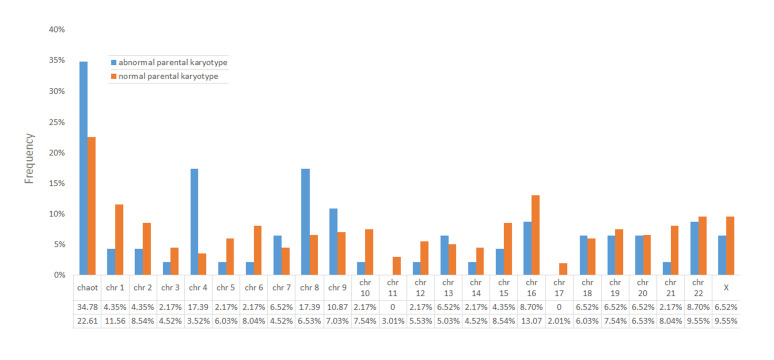
PGS-array-CGH and embryos chromosomal abnormalities frequency. PGS; Pre-implantation genetic screening and Array-CGH; Array comparative genomic hybridization.

Based on the PGS-array-CGH results, of 13 abnormal karyotype couples, 20.68% (12/58) of analyzed embryo were normal and all of them were transferred in 9 cycles. Finally, 33.33% (3/9) led to pregnancy and ended to live births. In the 70 normal karyotype couples, 70 cycles PGS-array-CGH were performed, and 29.92% (85/284) of embryos were normal. In 72.85% (51/70) of cycles, embryo transfers (ETs) were carried out and 52.94% (27/51) of ETs lead to successful pregnancy. Noticeably, 70.37% of pregnancies was led to live births (Fig .1,  Table S1B, See Supplementary Online Information in www.ijfs. ir). The frequency of chromosomal abnormalities in PGSarray-CGH embryos is shown in Figure 2.

Totally, 46 abnormal embryos were developed from abnormal karyotype couples; which among these, 16 embryos (16/46-34.78%) showed a chaotic chromosomal complement. Abnormality in chromosomes 17 and 11 was not observed in the embryos. Also, 199 abnormal embryos were obtained from normal-karyotype couples; the high rate of chaotic embryos is significant (45/199- 22.61%). Also, the lowest frequencies were related to abnormality in chromosomes 17 (4/199-2.01%) and 11 (6/199-3.01%).

## Discussion

RPL is a multifactorial problem. Several studies were conducted to examine the prevalence of RPL risk factors (115-17). In this study, we evaluated five RPL associated factors, including chromosomal abnormality, anatomical character, endocrine, thrombotic defects and sperm DNA damages. Here, we observed high frequency of Endocrine disorder. Also, hypothyroidism was identified as the most common endocrine disorder, consistent with some previous reports (17, 18). The incidence of chromosomal abnormalities was 15.61%, which is inconsistent with previous studies. This different frequency was observed probably because of the variety in sample size and nationality (19, 20). Here, we observed translocation as a most common abnormality that is consistent with other investigations (21, 22). Noteworthy, the chromosome 9 inversion was the most frequent structural chromosomal abnormality in the present study of, it is associated with reproductive complications as described previously (23, 24). Although, Merrion and Maisenbacher (25) denied this association.

PGS technology has improved the IVF success rate by improving embryo selection for transfer and subsequently, reducing pregnancy loss. Recent molecular cytogenetics development, such as FISH and array-CGH, have provided a rapid embryonic chromosomes screening tool at the preimplantation stage (26). Because of some limitations, only small numbers of our participants could benefit of PGS service.

Chromosome 16 disruption was observed more than other chromosomal abnormality in embryos of the normal karyotype parents. It is consistent with previous studies (21, 27).

## Conclusion

Clinical examination of a large proportion of Iranian couples with RPL history, indicated that hypothyroidism, anatomic factors and chromosomal anomalies are the major risk factors for RPL phenotype. Therefore, assessment of the mentioned factors would be useful for early diagnosis of RPL patients. Furthermore, identification of genetic causes of RPL could be considered to predict the risk of next pregnancy loss and would assist physicians for precise patient management in the clinic. Based on this retrospective study, it seems PGS platforms might provide a better chance for RPL couples.

## Supplementary PDF


