# Supplementary material for: Risk Factors Associated with Recurrent Pregnancy Loss and Outcome of Pre-Implantation Genetic Screening of Affected Couples
Source: Int J Fertil Steril. 2021 Oct 16;15(4):269–74. doi: 10.22074/IJFS.2021.137626.1027 (PMC8530214; doi:10.22074/IJFS.2021.137626.1027)
Supplement: Supplementary file 1 [file Int-J-Fertil-Steril-15-269-s01.pdf]

Int J Fertil Steril
International Journal of Fertility & Sterility
ISSN:2008-076X eISSN:2008-0778

Risk Factors Associated with Recurrent Pregnancy Loss and Outcome of Pre-Implantation Genetic Screening of Affected Couples

Nayeralsadat Fatemi, Ph.D.[1, 2#], Maryam Varkiani, M.Sc.[2, 3#], Fariba Ramezanali, M.D.[4], Babak Babaabasi, M.Sc.[2], Azadeh Ghaheri, Ph.D.[5], Alireza Biglari, Ph.D.[1*], Mehdi Totonchi, Ph.D.[2, 6*]

1. Department of Genetics and Molecular Medicine, School of Medicine, Zanjan University of Medical Sciences (ZUMS), Zanjan, Iran
2. Department of Genetics, Reproductive Biomedicine Research Center, Royan Institute for Reproductive Biomedicine, ACECR, Tehran, Iran
3. Department of Molecular Genetics, Faculty of Basic Sciences and Advanced Technologies in Biology, University of Science and Culture, Tehran, Iran
4. Department of Endocrinology and Female Infertility, Reproductive Biomedicine Research Center, Royan Institute for Reproductive Biomedicine, ACECR, Tehran, Iran
5. Reproductive Epidemiology Research Center, Royan Institute for Reproductive Biomedicine, ACECR, Tehran, Iran
6. Department of Stem Cells and Developmental Biology, Cell Science Research Center, Royan Institute for Stem Cell Biology and Technology, ACECR, Tehran, Iran

Table S1: PGS outcome in RPL couples

PGS/aCGH						
Chromosomal sate of couples	Cycles	Cycles with embryo transfer	Pregnancy	Pregnancy/Embryo transfer	L ive birth	Normal embryo/Total
Normal	70	51	27	27/51 (52.94%)	19 (70.37%)	85/284 (32.19%)
Abnormal	13	9	3	3/9 (33.33%)	3 (100%)	12/58 (20.68%)
Total	83	60	30	30/60(50%)	22(73.33%)	97/306(31.69%)

PGS; Pre-implantation genetic screening, RPL; Recurrent pregnancy loss, and aCGH; Array comparative genomic hybridization.

Received: 5/October/2020, Accepted: 30/January/2021
*Corresponding Addresses:
P.O.Box: 45139-53115, Department of Genetics and Molecular Medicine, School of Medicine, Zanjan University of Medical Sciences (ZUMS), Zanjan, Iran
P.O.Box: 16635-148, Department of Genetics, Reproductive Biomedicine Research Center, Royan Institute for Reproductive Biomedicine, ACECR, Tehran, Iran
Emails: biglari@zums.ac.ir, m.totonchi@royaninstitute.org
# The first two authors equally contributed to this work.
